# Nanodrug Delivery Systems Modulate Tumor Vessels to Increase the Enhanced Permeability and Retention Effect

**DOI:** 10.3390/jpm11020124

**Published:** 2021-02-14

**Authors:** Dong Huang, Lingna Sun, Leaf Huang, Yanzuo Chen

**Affiliations:** 1 Shanghai Key Laboratory of Functional Materials Chemistry, East China University of Science and Technology, Shanghai 200237, China; y53200022@mail.ecust.edu.cn (D.H.); y30191353@mail.ecust.edu.cn (L.S.); 2 Engineering Research Centre of Pharmaceutical Process Chemistry, Ministry of Education, Shanghai Key Laboratory of New Drug Design, School of Pharmacy, East China University of Science and Technology, Shanghai 200237, China; 3 Division of Pharmacoengineering and Molecular Pharmaceutics, Eshelman School of Pharmacy, University of North Carolina, Chapel Hill, NC 27599, USA; leafh@email.unc.edu

**Keywords:** nanoparticles, tumor vascular regulation, EPR effect, angiogenesis, blood perfusion, vascular permeability

## Abstract

The use of nanomedicine for antitumor therapy has been extensively investigated for a long time. Enhanced permeability and retention (EPR) effect-mediated drug delivery is currently regarded as an effective way to bring drugs to tumors, especially macromolecular drugs and drug-loaded pharmaceutical nanocarriers. However, a disordered vessel network, and occluded or embolized tumor blood vessels seriously limit the EPR effect. To augment the EPR effect and improve curative effects, in this review, we focused on the perspective of tumor blood vessels, and analyzed the relationship among abnormal angiogenesis, abnormal vascular structure, irregular blood flow, extensive permeability of tumor vessels, and the EPR effect. In this commentary, nanoparticles including liposomes, micelles, and polymers extravasate through the tumor vasculature, which are based on modulating tumor vessels, to increase the EPR effect, thereby increasing their therapeutic effect.

## 1. Introduction

Solid tumors are the major cause of death worldwide and their treatment remains a challenge [1,2,3]. Chemotherapy is one of the few treatment options available for metastasized tumors which cannot be removed surgically; however, the effectiveness of this therapeutic modality is not yet satisfactory [4]. This problem mainly stems from the lack of tumor selectivity by these agents; hence, the occurrence of severe adverse effects limits the usage of chemotherapy [5]. Nanomedicines have been designed to guide drugs more precisely to tumor cells and away from sites of toxicity. These agents have numerous theoretical advantages over low-molecular-weight drugs, including high drug loading, specific targeting, and the ability to protect the payload from degradation and release the drug in a controlled or sustained manner [6]. Theoretically, nanomedicines with larger particle size leak more slowly from blood vessels compared with most chemotherapy drugs. Fortunately, vascular leakage is a major feature of the vasculature of solid tumors. Specifically, tumor neovasculature has larger lumens and wider fenestrations (200 nm to 1.2 μm in diameter) due to its lack of a smooth muscle layer and pericytes [7]. When injected intravenously, nanomedicines ranging in size from 10 to 500 nm tend to circulate for a long time and can preferentially access the tumor tissue through the leaky tumor vasculature; subsequently, they are retained in the tumor bed due to reduced lymphatic drainage [8,9,10,11,12]. This pathophysiological phenomenon based on abnormal tumor angiogenesis to increase the delivery of nanomedicines in tumors is known as “the enhanced permeability and retention” (EPR) effect [10,11,12,13]. Matsumura and Maeda first reported the EPR effect in 1986 [11]. Follow-up studies rigorously verified that the EPR effect can be observed using macromolecules with an apparent molecular size >45 kDa (the threshold for renal clearance) and a longer plasma half-life. In recent years, Ding et al. conducted real-time research on human kidney tumors using X-ray computed tomography to confirm the existence of the EPR effect in humans. The results showed that the significant EPR effect can be found in >87% of human kidney tumors [14]. However, low-molecular-weight contrast agents do not stay in the tumor and can be washed out in a minute from tumor, which greatly differs from macromolecular drug retention in tumors. Therefore, Maeda et al. reported a more distinct method to prove the EPR effect in human by conjugating lipiodol with a macromolecular nanodrug [15]. This method lasts longer than X-ray computed tomography, and it can be used to further explore the significant difference between the EPR effect of macromolecular drugs and low-molecular-weight counterparts.

Nanodrug delivery is based on the accumulation of drugs in tumors due to the EPR effect, and the subsequent release of the therapeutic payload [11,16]. However, the EPR effect is inadequate in tumors; this inadequacy can be attributed to the high interstitial fluid pressure (IFP), the dense extracellular matrix (ECM), and the occluded or embolized tumor blood vessels [12,17,18]. Moreover, the prolonged circulation of the drug increases the ability of extravasation into the tumor through the EPR effect. Clinically, it has been demonstrated that the function of long-circulating liposomes, for example, doxorubicin (DOX)-loaded polyethylene glycol (PEG)ylated liposomes (Doxil), reduces opsonization and premature clearance, increases the blood circulation time, and potentially enhances drug accumulation in the tumor [19]. However, when the EPR effect is insufficient, the drug may extravasate and bring more toxicity into normal tissues. Thus, there is an urgent need to identify the physiological barriers that affect the EPR effect of tumors. The aim of such research would be the development of methods to enhance tumor penetration and retention, thereby improving tumor targeting and the therapeutic effect. In this review, we analyzed the barriers to drug delivery, focusing on the influence of tumor vasculature on the EPR effect. Moreover, we discussed the method utilized for the regulation of tumor blood vessels through the nanodrug delivery system to enhance the EPR effect [20,21,22,23,24].

## 2. Abnormal Vascular Functions Affect the Tumor EPR Effect

To satisfy the overgrowth of tumor cells, solid tumors need to induce and maintain a dedicated tumor blood supply, which is termed neovascularization. Under inflammatory or hypoxic tumor conditions, cells such as vascular endothelial cells release vascular permeability mediators, resulting in more enhanced tumor vascular permeability than in normal tissue, which can be demonstrated by angiography [25]. However, due to their short half-life and the rapid dilution in the bloodstream, these mediators mainly affect tumor vessels, but not normal tissue blood vessels. In such regions, macromolecules ranging from 10 to 500 nm (e.g., macromolecular anticancer agent, albumin, immunoglobulin, micelles, liposomes, and protein–polymer conjugates) can selectively leak out from the vascular bed and accumulate inside the interstitial space. However, in solid tumors, the EPR effect exhibits great heterogeneity. Tumors show different EPR effects regardless of their types and sizes, patients, or their developmental stages. Tumors with high blood vessel density (e.g., hepatocellular carcinoma) show a strong EPR effect, whereas others with low vascular density (e.g., pancreatic cancer) show a weak EPR effect [5]. Therefore, accurate monitoring and evaluation of the EPR effects in different tumors is essential for the development of personalized EPR-mediated plans for the treatment of tumors.

In principle, due to the widespread presence of EPR in tumors, nanomedicines based on the EPR effect show great promise for improving the efficacy of systemic anticancer drug therapy. However, their full anticancer potential has been hindered because of biological and pathophysiological barriers [26]. Obviously, the vascular system of tumors, which exhibit different vessel density, maturity, perfusion, and pore cutoff size, could be considered one of the main factors that affect the EPR effect [27]. In this review, we summarize the three main approaches through which abnormal tumor blood vessels affect the EPR effect and the related vascular mediators (Table 1).

### 2.1. Abnormal Angiogenesis

Angiogenesis is essential for the continuous growth and development of solid tumors. Tumor vessels provide oxygen and nutrients and remove waste products, supply a favorable niche for cancer stem cells, and serve as a conduit for tumor cell metastatic spread and immune cell infiltration. Unlike normal blood vessels, tumor blood vessels with abnormal structure and function impede the delivery of adequate and effective oxygen, as well as therapeutic drugs to cancer cells [88,89]. In cancer progression, the overexpression of proangiogenic factors drives the pathological angiogenesis. An imbalance between local proangiogenic and antiangiogenic factors may lead to the proliferation, migration, and new vessel formation of endothelial cells (EC). Furthermore, pericyte coverage of EC is often absent in the tumor vasculature. Compared with normal tissue with an organized microvasculature with regular branching order, the vascular organization of tumor tissue is disorganized and lacks the conventional hierarchy. Abnormal angiogenesis may lead to structural and functional abnormalities of the vascular system, which are often characterized by tortuous, unorganized, and excessive leakage [90,91]. This feature contributes to the vascular permeability of fluids and the escape of metastatic cancer cells [92,93]. Furthermore, the solid pressure generated by the proliferation of cancer cells compresses the blood and lymphatic vessels in the tumor, further impairing blood and lymphatic flows. These abnormal vascular structures collectively lead to an abnormal tumor microenvironment (TME), characterized by high IFP, hypoxia, and acidosis [88,94,95]. A physiological consequence of these vascular abnormalities is heterogeneity of tumor blood flow, which can interfere with the EPR effect and the uniform distribution of drugs within the tumor.

Tumor cells can promote blood vessel sprouting by releasing angiogenic molecules that bind to their respective receptors in adjacent cells or by paracrine signals [96,97]. Vascular endothelial growth factor (VEGF) appears to play the most critical role in physiological and pathological angiogenesis among all the known angiogenic molecules. It is overexpressed in the majority of solid tumors [28,29] and can promote the survival and proliferation of ECs, increase the display of adhesion molecules on these cells, and increase vascular permeability. By downregulating VEGF signaling in solid tumors, the vasculature may return to a more “normal” state, accompanied by decreased IFP, increased tumor oxygenation, and improved drug permeability in these tumors [98].

In addition to VEGF, other factors and proteins can also promote the abnormal formation of tumor blood vessels. Thus far, 28 proangiogenic factors/genes have been found to mediate tumor angiogenesis [76,77], including the fibroblast growth factor (FGF), hypoxia-inducible factor (HIF), platelet-derived growth factor-B (PDGF-B), tumor necrosis factor-α (TNF-α), chemokines, integrins, and transforming growth factor-β (TGF-β), as well as their receptors [76,99,100,101,102,103]. Acidic and basic FGF (FGF1 and FGF2) have the ability to induce angiogenesis [39]. FGFs stimulate the proliferation and migration of ECs, as well as the production of collagenase and plasminogen activator (PDGF), which stimulate angiogenesis and are related to the aging process of the tumor vasculature in vivo [42,43]. TGF-β possesses dual pro- and antiangiogenic properties. At low levels, TGF-β participates in the switch of angiogenesis by upregulating angiogenic factors and proteinases. At high levels, it can inhibit EC growth, stimulate the differentiation and recruitment of smooth muscle cells, and promote the reorganization of the basement membrane [52]. Moreover, as effective angiogenic factors, chemokines can induce the migration and proliferation of ECs, and they have pro- or antiangiogenic activities [104]. As an angiogenic factor, HIF cooperates with TNF inhibitors to initiate angiogenesis under hypoxic conditions [48,49,50,51]. It activates the signaling pathway and upregulates the expression of VEGF. Growth factors generated by this pathway activate the mitogen-activated protein kinase and protein kinase B signaling pathways, leading to increased levels of HIF-1 protein, thereby promoting tumor angiogenesis. Adhesion molecules (e.g., α_6_β_1_ and α_6_β_4_ integrins) mediate VEGF-induced angiogenesis, which regulates the adhesion of ECs to the ECM, thereby promoting the migration and survival of tumor vasculature. Other integrins (e.g., α_v_β_3_, α_v_β_5_, and α_5_β_1_) also mediate angiogenesis [63,64].

### 2.2. Irregular Blood Flow

Compared with normal vessels, newly formed tumor vessels are irregular or inconsistent [87]. It has been reported that tumor vessels are insensitive to angiotensin receptor type 2 (AGTR2). In addition, there is intermittent flow (only one flow in 15–20 min) and reverse flow of blood at the tumor site [105,106]. Moreover, blood often flows in the opposite direction. Irregular blood flow in the tumor is usually caused by irregular vascular structure. Unlike normal tissues, angiogenic factors in tumors at the late stage of vascular maturation will continue to be activated, leading to vascular abnormalities, which are characterized by irregular vascular structure and spatiotemporal heterogeneity [107]. Tumor vessels with irregular structure are characterized by a curved vascular shape, filling of the EC septum, and damage of the basement membrane. These effects lead to distortion of the vascular morphology and high permeability of the vascular EC space [31,108,109,110]. The distortion of blood vessels increases the geometric resistance of blood flow. The high permeability of blood vessels increases the hematocrit of tumor blood, thus increasing the blood viscosity [111]. In addition, the phenomenon of rapid proliferation of tumor cells in a finite space and excessive deposition of ECM can lead to large solid stress between adjacent cells and matrix components. The continuous accumulation of solid stress can lead to the compression of tumor blood vessels and the reduction of cross-sectional area and pressure difference in the direction of blood vessels [112]. The increase in vascular resistance and blood viscosity and the compression of accumulated solid stress significantly increases the resistance to blood perfusion. The increased resistance of tumor vessels to blood perfusion results in a low blood perfusion rate and a slow blood flow rate [113]. The change in blood flow velocity on the transport of nanoparticles through blood vessels has been investigated. A computer simulation explained the effect of blood flow velocity on the transport of nanoparticles. The results showed that the pressure at the vessel wall and the pressure gradient between the vascular wall and interstitial tissue increase in turn with the increase of fluid velocity in the vascular domain. Moreover, the trans-vascular transport efficiency of nanoparticles initially increases and subsequently decreases [114]. In addition, driven by the difference in pressure along the vascular direction, blood perfusion has the characteristics of convection–diffusion. Convection–diffusion differs between tumor blocks and depends on the local pressure gradient and flow resistance due to the heterogeneity of tumor blood vessels [115].

In addition to an irregular structure, the abnormal blood vessels of tumors also exhibit spatiotemporal heterogeneity [116,117]. This heterogeneity indicates the differing distribution of tumor vessels in various parts of the tumor or during the proliferation period. This is mainly indicated by the fact that the distribution of vessels in the periphery of the tumor is usually very rich, while their extension into the interior of the tumor gradually decreases. Therefore, this uneven distribution complicates the delivery of nanodrugs to the tumor center, which seriously hinders the penetration and extravascular transport of such agents. Of note, the high heterogeneity of tumor vessels in experimental mice and humans reduces the antitumor effects of some nanomedicines [26,118].

### 2.3. Extensive Vascular Permeability

Increased vascular permeability is widely found in endothelium discontinuous tumor vessels such as neovessels and immature vessels, as well as in other pathological tissues with disturbed vascular function. Compared with normal blood vessels, macromolecular drugs can reach the tumor stroma through the leaky vessel wall with large pores without hindrance [12]. However, excessive vascular leakage can cause plasma escape and hemoconcentration. This results in flow stasis and high IFP, which greatly hinder the extravasation of drugs and their movement to the tumor parenchyma. Furthermore, deposited clots of fibrin transiently promote the formation of blood vessels and ECM and prevent the penetration of antitumor therapeutic agents. The vascular media affecting the tumor vascular permeability are summarized below.

Bradykinin (BK) is of great importance in elevating the permeability of inflammatory sites and tumor tissues, thereby maintaining tumor growth [79,81]. Overexpression of BK receptors in solid tumors has been observed, resulting in defective vascular architecture with large intracellular gaps [119]. Kinin can activate EC-derived nitric oxide (NO) synthase, leading to increased levels of NO, a well-established and effective endothelium-derived vascular modulator [85,120,121]. NO is of great significance in vascular permeability, cell proliferation and extravasation (EPR effect), blood vessel dilation, and elevation of blood flow [83,84]. For example, NO generated from l-arginine under the action of NO synthase induces tumor vascular permeability. It has been demonstrated that the inhibition of NO generation can decrease vascular permeability, thereby weakening the EPR effect. This further confirms that NO is inextricably linked to vascular permeability in solid tumors [84,85]. Prostaglandins E1 and I2 are commonly involved in inflammation and cancer, exert similar effects to those of NO, and can enhance extravasation and EPR effects [83,86]. In summary, vascular permeability in tumors is often directly or indirectly related to kinins.

In addition, it has been shown that several vascular mediators, such as vascular permeability factor (VPF), which is important in tumor angiogenesis, TNF-α, and others elevate the vascular permeability of tumors [31]. EC survival and vascular permeability are closely related to the level of VPF/VEGF, as increasing this level can lead to upregulation of the corresponding receptors on ECs. [34,35]. TNF-α, a multifunctional proinflammatory cytokine with vascular permeabilizing effects [22], can enhance vascular leakiness via disrupting the EC adherence junction vascular endothelial cadherin [36]. TNF-α can increase the sensitivity to nanoparticles through serving as a vascular disrupting agent (VDA). At low levels, TNF-α may promote angiogenesis; however, at higher concentrations, it destroys the tumor vessels and increases the accumulation of drug in tumors [122].

## 3. Nanoparticles for Enhancing the Tumor EPR Effect

**Figure 1:**
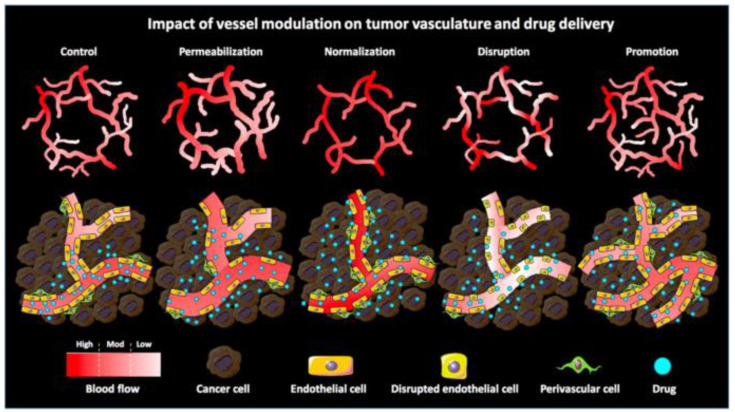
Schematic illustration of the impact of pharmacological vascular regulation strategies on tumor vasculature and tumor-targeted drug delivery. Vascular permeability enlarges the gap between ECs by vasodilating and increasing the gap between ECs and perivascular cells. Vascular normalization promotes vascular maturation and improves vascular perfusion, thereby restoring the morphology and function of tumor vasculature to a certain extent. Vascular rupture enhances vascular permeability by disrupting the endothelial lining while reducing perfusion (especially in immature vessels). Vascular facilitation increases relative blood volume in tumors by increasing vascular density and distribution. Reproduced from Ojha [20].

The EPR effect is an effective way for nanoparticles to passively target tumor cells. As opposed to passive drug targeting, nanoparticles based on the use of targeting ligands are termed “active drug targeting”. Actively targeted nanomedicines have failed to demonstrate benefit at the clinical level. This failure can be attributed to the fact that nanomedicines may face an insufficient endothelial vascular gap and a number of physiological barriers, such as high cellular density within solid malignancies and high IFP. Consequently, actively targeted nanoparticles have difficulties in identifying target cells due to the inadequate EPR effect. Therefore, enhancing the EPR effect through the use of nanoparticles can provide a better platform for subsequent treatment by elevating blood pressure, or conjugating with antibodies or EPR enhancers such as NO-generating agents. Several techniques have been employed to enhance the EPR effect, including the inhibition of angiogenesis, upregulation of tumor blood perfusion, and disruption of vascular or enhancement of vessel penetration to modulate the tumor vasculature [15,79,109,123,124]. Moreover, Ojha et al. described several pharmacological strategies for vascular regulation (Figure 1). Combined with nanoparticles, these strategies can enhance the EPR effect and improve treatment (Table 2).

### 3.1. Antiangiogenesis

VEGF, FGF and their receptors, matrix metalloproteinases (MMPs), tubulin, and integrins are closely related to tumor survival, migration, metastasis, and angiogenesis [49,142,143]. It has been reported that drugs targeting these factors can inhibit tumor angiogenesis, thereby increasing blood perfusion and reducing the IFP [21,98,144]. Antiangiogenic agents, to some extent, can restore the pressure gradient between the vascular wall and tumor interstitium. Subsequently, they decrease the blood flow stasis to allow more nanoparticles to penetrate the blood vessels and reach the interstitial tissue [29,98,145]. Hence, antiangiogenesis improves the delivery of the therapeutic entities via maintaining the integrity of the EPR effect and reducing the IFP. Numerous different types of nanoparticles have been extensively investigated to facilitate the delivery of antiangiogenic agents [62,125,127].

Several studies have shown the potential effectiveness of soluble VEGF receptors on inhibiting pathological tumor angiogenesis. Nanoparticles are able to carry VEGF inhibitors to vascular EC. These inhibitors block pathological angiogenesis and promote tumor cell apoptosis, thereby inhibiting tumor growth and metastasis. Although nanoparticles are potentially applicable to antiangiogenesis, better delivery carriers that can improve the targeting activity are urgently sought. The arginylglycylaspartic acid (RGD) peptide can specifically bind to the integrin receptor of tumor vascular ECs with high affinity [146,147]. Grafting RGD onto nanoparticles may improve their active targeting ability and increase the drug transfection efficiency under conditions of sufficient EPR. However, Storm et al. stated that the potential of RGD-conjugate tumor targeting should not be overestimated due to the RGD receptors being widely distributed on blood vessels, which can induce the less tumor selectivity [148,149].

Some RNA interference (RNAi) strategies that require entry into tumor cells to function, such as small interfering RNA (siRNA) and short hairpin RNA (shRNA), are ideal for tumor-specific VEGF inhibition. The strategy of silencing VEGF by RNAi has achieved satisfactory results in some solid tumor models [150,151,152,153,154]. The angiogenesis of VEGF is mediated by binding to two endothelium-specific receptor tyrosine kinases with high affinity, namely, FLT1 (VEGFR1) and FLK1/KDR (VEGFR2). The use of homologous tyrosine kinase receptor soluble FLT1 (sFLT1) gene therapy has illustrated that the transduced sFLT1 protein can bind to VEGF and inhibit its activity, and this binding is similarly characterized by high affinity. Kim et al. reported an angiogenic EC-targeted polymeric gene vehicle, polyetherimide-*g*-polyethylene glycol (PEG)–RGD, which contained sFLT1 protein and siRNA [125,126]. These nanoparticles can effectively transfer therapeutic genes to angiogenic ECs, but not to nonangiogenic cells, and effectively inhibit the proliferation of VEGF-responsive ECs by the delivered genes. Kanazawa et al. prepared the amphiphilic and cationic triblock copolymer as an siRNA carrier to efficiently deliver small interfering VEGF into tumor tissues and significantly inhibit tumor growth because of the suppression of VEGF secretion from tumor tissues [155].

Some other vascular mediators are also involved in tumor angiogenesis. Targeting these mediators can also effectively inhibit abnormal tumor angiogenesis. Endostatin, a peptide cleaved from the carboxy terminus of collagen XVIII, suppresses the cell cycle and expression of antiapoptosis genes in proliferating ECs, thereby suppressing angiogenesis. To assess the endostatin gene therapy, Oga et al. prepared polyvinylpyrrolidone–pentostatin nanoparticles which exhibit a strong antiangiogenic effect and effective inhibition of metastatic growth in the brain [62]. Moreover, the combined use of sFLT1 with endostatin could be an effective antiangiogenic approach to the treatment of unresectable hepatocellular carcinoma [156]. Pigment epithelium-derived factor is a type of glycoprotein that plays a universally acknowledged role in the inhibition of angiogenesis via downregulation of VEGF [65]. The cyclic RGD–PEG–polyetherimide exhibited increased gene transfection efficiency in human umbilical vein ECs via binding to α_v_β_3_, and significantly inhibited tumor growth and angiogenesis [157]. The binding of activated NF-κB to DNA can promote angiogenesis in addition to its role in facilitating cell proliferation, regulating apoptosis, facilitating angiogenesis, and stimulating invasion and metastasis [66]. Xiao et al. inhibited the growth and metastasis of breast cancer through delivering p65 shRNA into cells with a bioreducible polymer to block the signaling of NF-κB [158]. The proangiogenic effects of thyroid hormone on ECs and vascular smooth cells are initiated from the cell surface receptor for the hormone on the extracellular domain of integrin α_v_β_3_ [67]. Tetraiodothyroacetic acid (tetrac) is a deamination product of l-thyroxine that blocks thyroid hormone binding with the integrin receptor [159]. Therefore, tetrac combined with liposomes and poly(lactide-*co*-glycolic acid) nanoparticles can achieve tetrac targeting of cell membrane integrin α_v_β_3_ receptors and significantly inhibit angiogenesis [127,128,129,130]. MMPs participate in the process of angiogenesis in tissue reconstruction and neovascular growth through their proteolytic effect. Moreover, they release angiogenic factors residing in the matrix. Therefore, MMP inhibitors decrease angiogenesis and the migration of tumor cells, leading to slower progression of transplanted tumors [68]. Indeed, the antitumor efficacy of angiostatin and tissue inhibitor of metalloproteinases (TIMPs) has been demonstrated in various types of solid tumors [160,161]. Dendrimers containing plasmids of angiostatin and TIMP-2 showed high antitumor and antiangiogenic activity [162]. Nevertheless, antiangiogenic drugs also reduce the gap between tumor vascular ECs. Hence, the size of nanoparticles has to be strictly controlled if antiangiogenic drugs are employed to enhance the EPR effect [114]. 

### 3.2. Upregulated Tumor Blood Perfusion 

**Figure 2:**
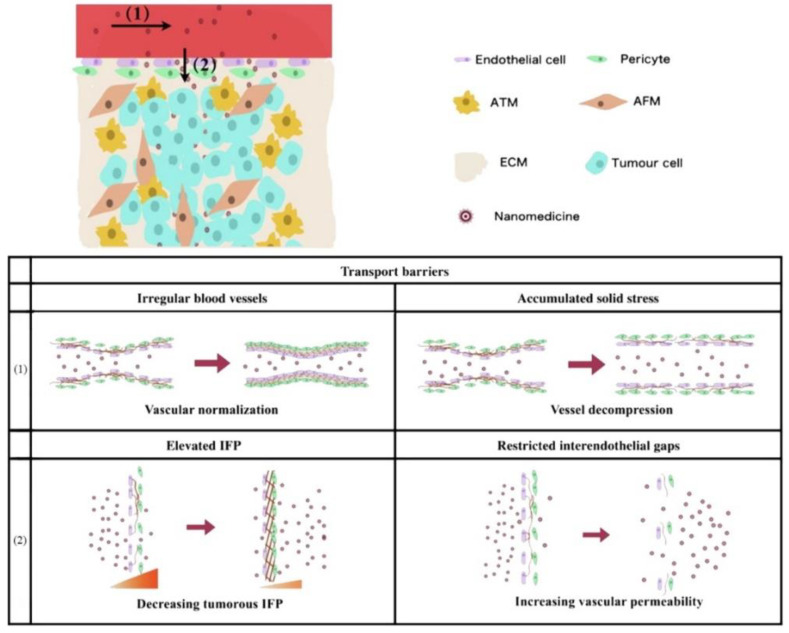
Schematic representation of vascular-related problems and countermeasures faced in the process of nanodrug penetration. (1) Intravascular transport: vascular irregularity and cumulative solid stress are resolved by normalization and decompression, respectively. (2) Trans-vascular transport: elevated interstitial fluid pressure (IFP) and limited endothelial space are improved by decreasing intertumoral IFP and increasing vascular permeability, respectively. Adapted from Yang et al. [115].

The main obstacle of blood perfusion in intravascular transport is due to irregular vascular structure and accumulated solid stress. Therefore, in accordance with the above two points, the blood perfusion of tumor vessels can be upregulated by vascular normalization and decompression, respectively (Figure 2). Yang et al. concluded that the former can use angiogenesis inhibitors to improve blood perfusion, so as to reduce the transport resistance of nanoparticles [115,163]. The latter can effectively reduce the solid stress through ablation of cells or the ECM, thus increasing the diameter of blood vessels to promote intravascular transport. Reduced blood flow directly limits the perfusion of nanoparticles into the tumor site [164]. In addition, the proliferating cancer cells in the center of the tumor tissue will form excessive pressure and compress the blood vessels and lymphatic vessels, leading to vascular collapse [88,94,95]. This results in an abundance and scarcity of functional blood vessels and lymphatic vessels in the periphery and center of the tumor, respectively [165]. This uneven distribution of blood vessels further worsens the relatively weak penetration ability of nanoparticles.

Vascular promotion is a vascular regulation strategy that addresses the issue of poor accumulation and distribution of drugs in tumors via increasing the vascular density and upregulating blood perfusion. Induction of angiogenesis appears to promote tumor growth. However, moderate induction of angiogenesis or vascular promotion may also contribute to better enrichment and distribution of anticancer drugs and improve their anticancer efficacy in some tumor models [20]. Among the recently developed strategies, the use of vasodilator-encapsulated nanoparticles for tumor angiectasis has been investigated as a potential option for promoting the extravasation of nanoparticles in tumors. Some vasodilator formulation nanoparticles have been employed, including angiotensin inhibitor, antihypertensive agents, gaseous vascular mediator-generating vasodilators, and ECM degradation agents.

The change of angiotensin I to angiotensin II mediated via carboxypeptidase can be inhibited by angiotensin-converting enzyme inhibitors (ACEI). AGTR2 is an effective agent in enhancing blood flow and promoting vascular permeability in tumors due to its vasoconstrictive function in healthy tissues, as well as increasing the systemic blood pressure. It has been shown that the perfusion of tumor vessels is gradually shifted from poor to good after slow systemic administration of AGTR2 [87]. An increase in BK levels leads to the activation of endothelial NO synthase. ACEIs (e.g., captopril) inhibit the degradation of BK, thereby increasing its local concentration in tumor tissues. Captopril, an ACEI, acts by downregulating the expression of AGTR2, thereby dilating blood vessels and lowering blood pressure. A combination of captopril with paclitaxel-loaded nanoparticles has been employed to simultaneously ameliorate tumor perfusion and expand EC gaps, thus enhancing nanodrug delivery to cancer cells [132]. Meanwhile, losartan is an angiotensin II receptor antagonist that increases nanodrug delivery through two mechanisms [166]. Losartan can lower solid stress that compresses blood vessels, thus improving vessel perfusion and drug delivery. However, it also increases the intratumoral penetration of the intraperitoneally or intravenously injected nanoparticles into the tumors by decreasing the ECM [167]. 

In addition to AGTR2, other drugs may also expand blood vessels. Hydralazine (HDZ), a drug applied to hypertension and heart failure therapy, has been used as a tumor vasodilator to modulate the TME. It is thought that HDZ functions by dilating blood vessels. Therefore, Chen et al. prepared HDZ-encapsulated liposomes which can expand tumor vessels and strengthen tumor permeability. These liposomes also ameliorated the accumulation and permeation of nanoparticles inside the tumor. Compared with free HDZ, intravenous injection of these liposomes in desmoplastic tumor-bearing mice prolonged the blood circulation time of HDZ. Moreover, its vasodilation effect increased the penetration and accumulation of nanoparticles into tumors mediated by the EPR effect to some extent [131]. Of note, in vivo and in vitro studies have shown that HDZ exerts certain antiangiogenesis effects [168]. Therefore, such nanomedicines have great potential in upregulating tumor blood perfusion. Sildenafil, a conventional medicine utilized for pulmonary hypertension therapy, can be utilized for developing effective and tumor-selective angiectasis approaches. Sildenafil can be encapsulated into the hydrophobic core of a cisplatin-incorporated polymeric micelle to form a nanoparticle with a hydrophobic center and a dense PEG shell. This polymeric micelle is effective in dilating tumor vessels and boosting the accumulation of cisplatin–sildenafil coloaded nanoparticles in tumors [133].

Endogenous signal molecule endogenous carbon monoxide (CO) and heme oxygenase (HO) play an important role in regulating vascular tension and inducing angiogenesis [69,70]. Fang et al. clearly demonstrated that vascular permeability and blood flow were significantly increased after using CO donors or HO-1 inducers (PEGylated heme) [72]. They designed two CO generators with tumor selectivity. The first was the CO external donor tricarbonyldichlororuthenium (II) dimer nanomicelle, which can slowly enhance the release characteristics and selective accumulation of tumors mediated by the EPR effect [71]. The second was the HO-1 inducer (PEGylated hemin), which can be selectively enriched in tumors after injection and produce CO by inducing HO-1 expression in tumors [72,73]. In solid tumor models, both nanodrugs exhibited higher selectivity for CO production in tumor tissues versus normal tissues, which resulted in augmented tumor blood flow recovery [72]. 

Platelets preserve tumor vessel integrity and prevent nanomedicines from diffusing into solid tumors. Previous findings have shown that the specific depletion of tumor-associated platelets may be a potent approach for disrupting vascular barriers and enhancing the extravasation of nanoparticles from tumor vessels [169,170,171]. Nie et al. showed that drug delivery can be facilitated via functionalizing nanoparticles, thereby locally depleting tumor-associated platelets that normally restore the leaky vessels [172]. They developed a polymer lipid peptide nanoparticle core consisting of a charged amphiphilic polymer where the positively charged region adsorbs the antibody R300. The platelet-specific R300 antibody can bind to platelets, leading to their micro-aggregation and subsequent removal by macrophages, and further increasing the intratumoral accumulation and retention of drugs.

**Figure 3:**
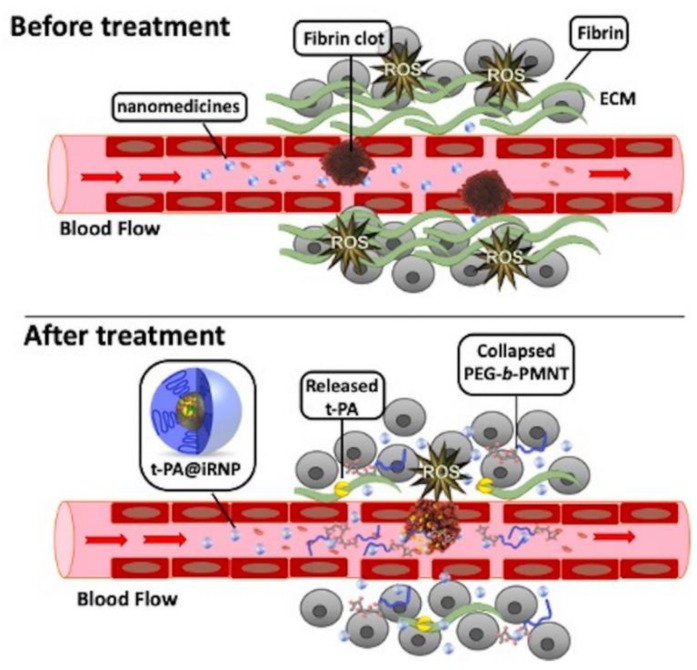
Schematic representation of transmission and action in the microenvironment of large amounts of fibrin around colon tumors and their tissues. Compared with free tissue plasminogen activator (t-PA), using t-PA-assembled redox-active nanoparticles (T-PA@iRNP) can effectively relieve the compressed tumor vessels and upregulate blood perfusion, and improve the poor distribution of nanodrugs in tumors. Reproduced from Mei [134].

Excessive constituents of ECM, such as collagen, fibrin, laminin, elastin, and aggregated platelet in the TME, deposit in the tumor vessels [173]. This hinders the blood supply, impairing the delivery of drugs to the tumor site and reducing efficacy. However, degradation of ECM components by enzymatic treatment (e.g., collagenase) can improve the vascular properties and upregulate blood perfusion at tumor sites [174,175]. Tissue plasminogen activator (t-PA) binds to drug carriers to degrade fibrin. Mei et al. developed t-PA-assembled redox-active nanoparticles (T-PA@iRNP) by degrading fibrin to reduce the pressure on tumor blood vessels, thereby increasing the perfusion of blood and nanomedicines in tumors (Figure 3). When applied to colon cancer models, T-PA@iRNP degradation of deposited fibrin enhances the infiltration of iRNP and immune cells into tumor tissues through an increase in blood flow. This enhances the EPR effect and consequently amplifies the inhibitory effect on tumor growth [134]. Zhang et al. encapsulated DOX and near-infrared spectroscopy-activated losartan in hollow mesoporous Prussian blue nanoparticles to degrade the ECM. The results showed that losartan-containing nanoparticles can enhance the penetration of DOX, and exhibit a good tumor inhibition effect under the synergistic action of photothermal therapy/chemotherapy [135].

### 3.3. Enhanced Vessel Penetration

The gap in tumor vascular ECs is one of the important bases of the EPR effect. However, in some tumors with poor permeability, the size and rate limitation of large-scale nanodrugs by the vascular endothelial space cannot achieve the purpose of trans-vascular transport [115]. Therefore, augmenting the permeability of tumor vessels and even destroying the vascular system can effectively promote extravasation [176,177]. Nanodrugs with a size <10 nm can effectively permeate inside tumors via trans- and extravascular transport. However, the rapid clearance by the kidneys is a problem, resulting in an insufficient EPR effect. Nanodrugs, with size ranging 50–200 nm, can realize long-time circulation and passively target the tumor site by intravascular transport. However, due to the existence of various barriers, they often have difficulty in reaching the core of the tumor. Therefore, nanodrugs of variable size can be used to simultaneously achieve long-time circulation, good passive targeting, and high permeability [178,179,180,181,182,183].

Integrating VDAs in nanomedicines is a promising therapy for meliorating vascular permeability and the EPR effect. Several VDAs have been evaluated; for example, combretastatin A4 phosphate (CA4P) is a tubulin-binding agent which induces vessel disruption by suppressing tubulin polymerization. Furthermore, flavonoid acetic acid-based agent 5,6-dimethylxanthenone-4-acetic acid (DMXAA) increases the levels of NO and serotonin, resulting in weak endothelial function. Sengupta et al. introduced poly(lactide-*co*-glycolic acid) nanoparticles conjugated to DOX, which were trapped in a phospholipid block-copolymer membrane containing CA4P [136]. The nanoparticles were designed to first release CA4P, which initially induces vessel disruption, thereby creating a niche for the release of DOX. This approach was linked to significant tumor inhibition and improvement in overall survival. VDAs and other physiological agents are commonly used to enhance vascular permeability and, thus, promote the extravasation of nanoparticles. Zhang et al. developed a bioinspired nanodesign, which combined vasculature-destructive DMXAA and hypoxia-activated tirapazamine with a mesoporous silica nanoparticle core, as well as a hidden platelet membrane shell [137]. The platelet membrane can be continuously “recruited” by the tumors with characteristics of artificial blood vessel destruction. The results indicated that disruption of the tumor vasculature caused by DMXAA and the platelet membrane-mediated targeting of the intratumoral disrupted vasculature were beneficial to each other and strengthened mutually. Studies have shown that the EPR effect of nanoparticles is induced by rupture of blood vessels, which is closely related to tumor density and the speed of blood flow [176]. As mentioned above, the tumor vasculature consists of only a single layer of ECs with a missing or incomplete basement membrane [184]. Furthermore, the vasculature is closely related to the blood supply of tumor cells [185,186]. Destruction of the vasculature can significantly improve the EPR effect and, if the vasculature is inadequate, the tumor tissue will undergo programmed death [187,188,189].

**Figure 4:**
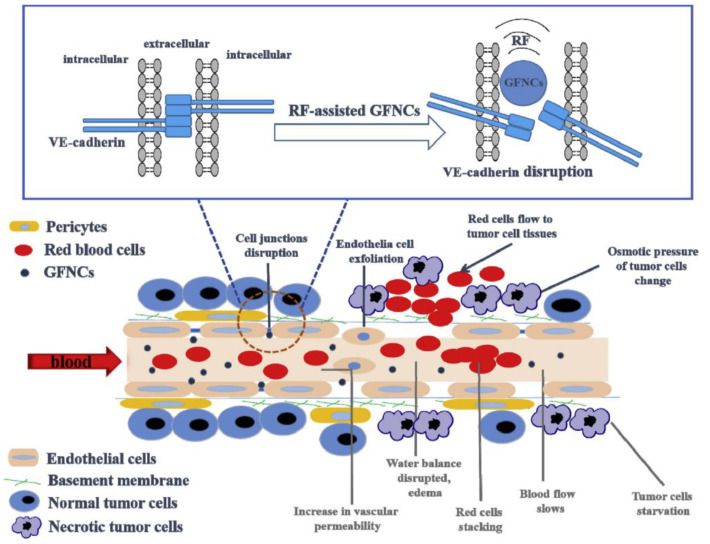
Schematic diagram of the mechanism of tumor vascular rupture after radiofrequency-assisted gadofullerene nanocrystal (GFNC) treatment. GFNC particles injected intravenously into tumor-bearing mice penetrate the vulnerability of tumor vascular ECs. When radiofrequency irradiation is applied, the sudden volume expansion of GFNCs can lead to the destruction of vascular endothelial cadherin at the junction of endothelial adhesion bodies of tumor vessels, thereby increasing vascular permeability and realizing the destruction of tumor vessels. Reproduced from Li and Zhen et al. [139,190].

The development of strategies for interacting with ECs or destroying vascular EC connections is another effective approach to improving vascular permeability. Inspired by this, Palomba et al. transferred the purified leukocyte membrane onto nanoporous silicon particles to produce a type of leukolike vector (LLV) [138]. Multiple receptors on LLV can interact with ECs and reduce the vascular barrier function. The investigators also demonstrated that the leukocyte plasma membrane on the surface of LLV can effectively interact with the overexpressed intercellular adhesion molecule-1 (ICAM-1) in the tumor vasculature, activate the endothelial receptor ICAM-1 pathway, and boost vascular permeability through the phosphorylation of vascular endothelial cadherin. Li et al. found that phase-induced size expansion through radiofrequency-assisted gadofullerene nanocrystals (GFNCs) can destroy abnormal tumor vasculature. Biocompatible GFNCs with a nanoparticle size were designed to penetrate the leaking tumor blood vessel. With the assistance of radiofrequency, the phase transition occurs when GFNCs spill over the tumor vessels. In addition, the abrupt and drastic changes in nanoparticle structure caused by phase transition directly disrupt the abnormal tumor blood vessels (Figure 4). Treatment with this method can cause rapid ischemia, necrosis, and atrophy of tumor tissues, while significantly reducing the toxic and side effects of other antivascular treatments [139,190].

In addition to chemotherapy, some physical therapies can also significantly enhance blood vessel penetration and improve the effects of antitumor treatment. Ionizing irradiation can increase vascular leakiness by inducing EC apoptosis and enhancing the expression of VEGF and FGF [191]. Liang et al. designed a radioisotope therapy by encapsulating the radioisotope iodine-131 (^131^I)-labeled bovine serum albumin (BSA) in liposomes. ^131^I-BSA-liposomes were intravenously injected into 4T1 tumor-bearing mice. Compared with untreated mice, those treated with ^131^I -BSA-liposomes showed high retention in the tumor site, demonstrating enhanced tumor vascular permeability and improved EPR effect [140]. Koukourakis et al. underlined the value of combining radiotherapy with drug delivery systems based on nanomedicines [192]. Patients were treated with radiolabeled PEGylated liposomal DOX, and achieved an overall remission rate >70%. This is an effective anticancer treatment modality for inducing hyperthermia in tumors. This generally leads to an increase in blood flow and vascular permeability in tumors, thus promoting drug and oxygen supply to tumors [193]. Hyperthermia can be applied to increase the EPR effect, particularly in nonleaky tumors with low baseline levels of nanomedicine accumulation [194]. Temperature-sensitive liposomes have developed into an ideal nanocarrier for coadministration with hyperthermia, enabling triggered drug release locally at the heated tumor site. Several studies have demonstrated that drug delivery and intratumoral distribution can be ameliorated through combining temperature-sensitive liposomes with modest hyperthermia. It was found that the human ovarian carcinoma tumor model was rather impermeable to liposomes with a size of 100 nm at room temperature. However, as the temperature increased, the release of liposomes was significantly elevated [195]. Manzoor et al. established temperature-sensitive liposomes containing DOX, which can enhance blood vessel penetration and liposome accumulation [141].

## 4. Conclusions and Future Perspectives

The EPR effect, which involves the pathophysiological mediators and unique anatomical architecture of tumor tissues, is becoming a promising avenue for targeted anti-tumor therapy. Thus, the tumor-selective delivery of anticancer nanomedicines based on the EPR effect is becoming possible. However, the EPR effect can be highly heterogeneous. Specifically, in the complex tumor environment, it is difficult for nanoparticles to diffuse into vascular areas of the tumor due to high IFP, abnormal ECM, and massive interaction sites in the tumor. Hence, in the last couple of years, on the basis of the EPR effect, scientists have investigated other mechanisms of nanoparticle entry into solid tumors [196]. Recently, Sindhwani et al. proposed that most of the tumor vasculature is continuous and does not have sufficient EC gaps to explain the accumulation of nanoparticles in tumors. Moreover, they stated that most nanoparticles can reach the interior of the tumor via active trans-endothelial pathways rather than passive transport via gaps [197]. Although they found that the trans-endothelial pathways play a significant role in the accumulation of nanoparticles in tumor sites, their experimental method had certain limitations. Firstly, they only utilized PEGylated gold nanoparticles as simulated nanoparticles to examine the accumulation in the tumor, and could not cover the accumulation of other nanoparticles in tumors. Secondly, they used a Zombie mouse model to distinguish the contribution of the passive gap from active trans-endothelial transport. This model could deactivate active mechanisms and retain the passive way that fixed the mouse by transcardiac perfusion and relied on a peristaltic pump to retain a physiologically relevant flow rate. This could not simulate the blood vessels and blood flow under normal physiological conditions. Lastly, the blood driven by the peristaltic pump only circulated for a short period of time (15 and 60 min). In summary, trans-endothelial pathways may be a reason for the accumulation of nanoparticles in tumor sites; nevertheless, the EPR effect remains the basis of nanodrug delivery to tumors. Furthermore, nanoparticles which can improve tumor vessel penetration, reduce IFP, and degrade the ECM can be applied to enhance the EPR effect [26].

Herein, we summarized the mechanism of abnormal vascular functions, such as tumor angiogenesis, irregular blood flow, and extensive vascular permeability, as well as their influence on the EPR effect. In addition, we analyzed some nanoparticles developed to facilitate the EPR effect in tumors in response to the above factors. In terms of antiangiogenesis, gene therapy nanomedicines targeting angiogenic growth factors and their receptors are the most widely studied, and offer another approach to directly inhibiting tumor angiogenesis early in the process. Its diverse nanocarrier form provides a rich selection for delivery to different types of tumors. For irregular blood flow caused by abnormal vascular morphology and structure, blood perfusion can be effectively upregulated by slight vascular facilitation, vasodilation, or removal of excessive ECM in the TME. Nanoparticles encapsulated with different types of drugs exhibit the diversity and universality of nanocarriers, providing more possibilities for the selection of nanodrugs. However, EPR effect-based drug delivery strategies continue to be characterized by numerous problems and limitations. For example, enhancing the EPR effect may help maintain nutrient and oxygen transport, thereby accelerating tumor growth. Therefore, when designing such nanoparticles, it is particularly important to properly balance the relationship between tumor killing or inhibition and tumor growth promotion caused by the EPR effect.

## Figures and Tables

**Table 1:** Relationship between tumor vascular-related mediators and three typical vascular characteristics.

Features	Vascular Mediators	Functions	Tumors with This Substance	Reference
Abnormal angiogenesis	Vascular endothelial growth factor (VEGF)	Key factors in angiogenesis, VEGFs bind to the kinase function of VEGF receptor (VEGFR)-activated receptors, triggering a variety of downstream signaling cascades, such as increased capillary permeability, nitric oxide (NO) production (relaxation of vascular smooth muscle), endothelial cell (EC) proliferation, migration, and survival under stress.	Overexpression in most solid tumors	[28,29,30]
	Tumor necrosis factor (TNF)-α	TNF-α mediates monocyte differentiation into angiogenic cells that support tumor angiogenesis. It is also a multipotent proinflammatory cytokine with vascular permeability activity, which can enhance vascular leakage by disrupting the EC adhesion junction VE-cadherin.		[22,31,32,33,34,35,36]
	Acidic fibroblast growth factor (FGF)/FGF-1	Interacts with receptor tyrosine kinase subtypes to induce EC proliferation and maintain tumor angiogenesis.		[37]
	Basic FGF/FGF-2	Controls angiogenesis by inducing the expression of VEGF through paracrine and endocrine mechanisms.		[38,39,40]
	Platelet-derived growth factor (PDGF)	PDGF signals through two cell-surface tyrosine kinase receptors, PDGF receptor α (PDGFRα) and PDGFRβ, and induces angiogenesis by upregulating the production of VEGF and regulating the proliferation and recruitment of perivascular cells.		[41,42,43]
	Placenta growth factor (PLGF)	PLGF only binds to VEGFR-1 and induces tumor angiogenesis, promoting the survival of ECs in tumor-associated blood vessels.		[44]
	Epidermal growth factor (EGF)	A key EGF receptor (EGFR) ligand is one of many growth factors that drive the expression of VEGF.		[45]
	Hepatocyte growth factor (HGF)	Stimulates cell motility and the secretion of proteinases and plays an important role in tumor invasion and progression.		[46]
	Hypoxia-inducible factor (HIF)-1α	Upregulates VEGF gene expression by hypoxia response element binding to the promoter region of VEGF.		[47,48,49,50,51]
	Transforming growth factor (TGF) -β	Induces strong VEGF production in recruited hematopoietic cells, leading to activated angiogenesis and vascular remodeling. Low TGF-β levels contribute to angiogenesis, and high levels of TGF-β can inhibit EC growth.		[52,53,54]
	Interleukin (IL)-1β	Induces angiogenesis indirectly by activating the expression of VEGF in smooth muscle cells.		[55]
	IL-3	Stimulates EC movement and promotes the formation of new blood vessels in vivo. It also stimulates migration and proliferation of vascular smooth muscle cells.		[56]
	IL-6	Regulates the synthesis of VEGF and influences tumor angiogenesis by inducing the production of VEGF.		[57]
	IL-8	Enhances EC survival, proliferation, and matrix metalloproteinase production, and regulates angiogenesis.		[58]
	Neuropilin 1 and 2	Regulates receptor–ligand interactions of the VEGF family.		[59]
	Adrenomedullin	Promotes angiogenesis, protects cells from apoptosis and vascular injury, and affects vascular tone and permeability.		[60]
	Stromal cell-derived factor 1 (SDF-1), a chemokine	Synergizes with VEGF to induce angiogenesis in human ovarian cancer tumors. Furthermore, in invasive breast cancer, stromal fibroblast-derived SDF-1 promotes angiogenesis by recruiting bone marrow-derived endothelial precursors. It plays an angiogenic role through the receptor CXC motif chemokine receptor type 4.		[61]
	Endostatin	Inhibits cell cycle control and antiapoptotic genes in proliferating ECs, thus inhibiting angiogenesis.		[62]
	Integrin	Adhesion molecules such as α_6_β_1_ and α_6_β_4_ integrins mediate VEGF-induced angiogenesis, which regulates the adhesion of ECs to the ECM, thereby promoting the migration and survival of tumor vasculature. Other integrins (e.g., α_v_β_3_, α_v_β_5_, and α_5_β_1_) have also been shown to mediate angiogenesis.		[63,64]
	Pigment epithelium-derived factor	Inhibits angiogenesis via downregulation of VEGF.		[65]
	Nuclear factor kappa-B (NF-κB)	Activated NF-κB can bind to DNA, promote cell proliferation, regulate cell apoptosis, promote angiogenesis, and stimulate invasion and metastasis.		[66]
	Thyroid hormone	Thyroid hormones have proangiogenic effects on ECs and vascular smooth muscle cells initiated by integrin α_v_β_3_ extracellular domain hormone cell-surface receptors.		[67]
	Matrix metalloproteinases (MMPs)	Involved in the process of angiogenesis through its proteolytic role in tissue remodeling, as well as the growth of new blood vessels and the release of angiogenic factors sequestered in the matrix.		[68]
	Endogenous carbon monoxide (CO) and heme oxygenase (HO)	Play an important role in regulating vascular tension and inducing angiogenesis, and can significantly increase vascular permeability and blood flow.		[69,70,71,72,73]
	Angiogenin	Undergoes nuclear translocation in ECs where it stimulates ribosomal RNA transcription and cell proliferation.		[74]
	Angiopoietin 1	Activates matrix-degrading enzymes, including plasminogen activators and MMPs, to loosen the matrix and promote ECs migration.		[75]
	Vashohibin-1	A novel angiogenesis inhibitory protein regulates angiogenesis, inhibits pathological angiogenesis, and promotes tumor vascular maturation by negative feedback.	High expression in liver cancer, prostate cancer, renal cancer, and colorectal cancer	[76,77]
Vascular permeability	VEGF (VEGF-A/B/C/D)	As mentioned above.	Overexpression in most solid tumors	[28,29,30]
	Bradykinin (BK)	Activates EC-derived NO synthase, which leads to an increase in NO and plays a role in increasing vascular permeability.		[78,79]
	Hydroxyprolyl3 BK	As mentioned above.	Advanced cancer	[80,81,82]
	Inducible nitric oxide synthase (iNOS) and NO	NO is an effective endothelium-derived vascular regulator, which plays an important role in vascular permeability, cell proliferation, and extravasation (EPR effect), inducing vasodilation and increasing blood flow.		[83,84,85]
	Prostaglandin E1 and I2	Usually involved in inflammation and cancer, it has similar effects as NO and can enhance extravasation and EPR effects.		[83,86]
	TNF-α	As mentioned above.		[22,31,32,33,34,35,36]
	Angiotensin receptor type 2 (AGTR2)	AGTR2 can induce vasoconstriction in healthy tissues and increase systemic blood pressure, and is an effective substance to enhance blood flow and promote vascular permeability in tumors.		[87]
	IL-2	Increased vascular permeability by inducing NO production		[76,77]
	Endothelin-1 (Et-A, Et-B)	Endothelin is an endogenous long-acting vasoconstrictor regulator.		[76,77]
Irregular blood flow	AGTR2	As mentioned above.		[87]
	Endogenous CO and HO	As mentioned above.		[69,70,71,72,73]

**Table 2:** Nanoparticles for enhancing the tumor enhanced permeability and retention (EPR) effect.

Types of Nanoparticles	Size Range	Types of Tumor	Active Ingredients	Mechanisms for Enhancing EPR Effect	Reference
Polyetherimide–*g*–PEG–RGD	~200 nm	CT-26 colon adenocarcinoma	sFLT1 protein and siRNA	Inhibiting tumor-specific VEGF	[125,126]
Tetraiodothyroacetic acid (tetrac) combined with poly(lactide-*co*-glycolic acid) nanoparticles	~200 nm	Drug-resistant breast cancer orthotopic tumor	tetrac	Suppressing angiogenesis	[127,128,129,130]
Hydralazine (HDZ)–liposomes	88 ± 4 nm	Desmoplastic melanoma	HDZ	Expanding blood vessels	[131]
Captopril combined with paclitaxel-loaded nanoparticles	~100 nm	Human glioma (U87)	Captopril	Increasing tumor blood perfusion and enlarging endothelial gaps in tumor blood vessels	[132]
Cisplatin–sildenafil co-loaded nanoparticles	~200 nm	Murine melanoma	Sildenafil	Inducing vasodilation	[133]
Tissue plasminogen activator (t-PA)-installed redox-active nanoparticles	48 ± 2 nm	Mouse colorectal carcinoma	t-PA	Increasing drug delivery near the solid tumor through fibrin degradation and blood flow restoration.	[134]
(Losartan+ DOX) @ hollow mesoporous prussian blue nanoparticles	~187 nm	Mouse breast cancer	Losartan + DOX	Near-infrared spectroscopy-activated losartan in Prussian blue nanoparticles to degrade ECM, which improved the penetration of most nanoparticles	[135]
Nanocells (comprises a nuclear nanoparticle within an extranuclear PEGylated lipid envelope)	80–120 nm	Melanomas or Lewis lung carcinoma	DOX and combretastatin A4 phosphate	Enhancing vessel penetration	[136]
Platelet membrane-coated mesoporous silica nanoparticles (MTD@P)	~130 nm	Mouse colorectal carcinoma (CT26)	Tirapazamine and 5,6-dimethylxanthenone-4-acetic acid (DMXAA)	Increasing vascular permeability, through the tumor vessel disrupting effect of DMXAA.	[137]
Leukolike vector (LLV) modified nanoporous silicon particles	-	BALB/c 4T1 breast cancer	LLV	Activating the endothelial receptor intercellular adhesion molecule-1 (ICAM-1) pathway, and leading to increased vascular permeability through the phosphorylation of vascular endothelial cadherin	[138]
radiofrequency-assisted gadofullerene nanocrystals (GFNCs)	140–170 nm	Human liver hepatocellular carcinoma (HepG2-luc cells)	GFNCs	Significantly downregulating tumor vascular endothelial cadherin, leading to vascular collapse and destruction, thereby increasing vascular penetration	[139]
PEGylated ^131^I labeled bovine serum albu-min (^131^I-BSA)-liposomes	<200 nm	4T1 murine breast cancer	^131^I-BSA	Increasing vascular permeability by damaging tumor vascular ECs	[140]
Temperature-sensitive liposomes	~200 nm	Human squamous cell carcinoma and B16BL6 melanomas	DOX	Enhancing vessel penetration	[141]

## Abbreviations

ACEI	angiotensin-converting enzyme inhibitor
AGTR2	angiotensin receptor type 2
BK	bradykinin
BSA	bovine serum albumin
CA4P	combretastatin A4 phosphate
CO	carbon monoxide
DMXAA	5,6-dimethylxanthenone-4-acetic acid
DOX	doxorubicin
EC	endothelial cell
ECM	extracellular matrix
EGF	epidermal growth factor
EGFR	epidermal growth factor receptor
EPR	enhanced permeability and retention
Et-A/B	endothelin-A/B
FGF	fibroblast growth factor
FLK1/KDR	vascular endothelial growth factor receptor 2
FLT1	vascular endothelial growth factor receptor 1
GFNC	gadofullerene nanocrystal
HDZ	hydralazine
HGF	hepatocyte growth factor
HIF	hypoxia-inducible factor
HO	heme oxygenase
^131^I	iodine-131
ICAM-1	intercellular adhesion molecule-1
IFP	interstitial fluid pressure
IL	interleukin
iNOS	inducible nitric oxide synthase
LLV	leukolike vector
MMP	matrix metalloproteinase
NO	nitric oxide
NF-κB	nuclear factor kappa-B
PDGF	platelet-derived growth factor
PDGFR	platelet-derived growth factor receptor
PEG	polyethylene glycol
PLGF	placenta growth factor
RGD	arginylglycylaspartic acid
RNAi	RNA interference
SDF-1	stromal cell-derived factor 1
sFLT1	soluble vascular endothelial growth factor receptor 1
shRNA	short hairpin RNA
siRNA	small interfering RNA
tetrac	tetraiodothyroacetic acid
TGF-β	transforming growth factor-β
TIMP	tissue inhibitor of metalloproteinase
TME	tumor microenvironment
TNF-α	tumor necrosis factor-α
t-PA	tissue plasminogen activator
VDA	vascular disrupting agent
VEGF	vascular endothelial growth factor
VEGFR	vascular endothelial growth factor receptor
VPF	vascular permeability factor
